# Formation, Stability, and Crystallinity of Various Tricalcium Aluminate Polymorphs

**DOI:** 10.3390/ma17030735

**Published:** 2024-02-03

**Authors:** Simona Ravaszová, Karel Dvořák, Martin Boháč, Dalibor Všianský, Andrea Jančíková

**Affiliations:** 1 Faculty of Civil Engineering, Brno University of Technology, Veveří 331/95, 602 00 Brno, Czech Republic; ravaszova.s@fce.vutbr.cz (S.R.); janciku.a@fce.vutbr.cz (A.J.); 2 Research Institute for Building Materials, Hněvkovského 30/65, 617 00 Brno, Czech Republic; bohac@vush.cz; 3 Department of Geological Sciences, Faculty of Science, Masaryk University, Kotlářská 267/2, 611 37 Brno, Czech Republic; dalibor@sci.muni.cz

**Keywords:** tricalcium aluminate, polymorphism, cubic, orthorhombic, monoclinic, crystallite size

## Abstract

Tricalcium aluminate is an important phase of Portland clinker. In this paper, three polymorphs of C_3_A were prepared by means of the solid-state synthesis method using intensive milling of the raw material mixture which was doped with various amounts of Na_2_O and sintered at a temperature of 1300 °C for 2 h. The final products were evaluated through X-ray diffraction using Rietveld analysis. The effect of the Na dopant content on the change in the crystalline structure of tricalcium aluminate was studied. It was proven that the given preparation procedure, which differed from other studies, was close to the real conditions of the formation of Portland clinker, and it was possible to prepare a mixture of different polymorphs of calcium aluminate. Fundamental changes in the crystal structure occurred in the range of 3–4% Na, when the cubic structure changes to orthorhombic. At a dosage of Na dopant above 4%, the orthorhombic structure changes to a monoclinic structure. There are no clearly defined boundaries for the existence of individual C_3_A phases; these phases arise at the same time and overlap each other in the areas of their formation at different Na doses.

## 1. Introduction

Due to economic and environmental pressures, especially regarding the production of a lower volume of CO_2_, modern cement materials are increasingly complex from a chemical and mineralogical point of view [1]. The use of alternative fuels such as waste materials, biomass, and municipal solid waste can reduce our dependence on fossil fuels, reduce greenhouse gas emissions, and improve energy efficiency. However, this represents not only economic advantages but also technological challenges. This trend inevitably leads to a change in the properties of the individual phases of Portland cement. The properties of these components can therefore differ significantly from those expected for traditional Portland cement in previous years. This requires extensive research and testing [2,3,4,5,6].

Tricalcium aluminate (C_3_A) is one of the most important components of Portland cement (OPC). Tricalcium aluminate with a melting point of 1540 °C belongs to the group of the most represented calcium–aluminate phases in clinker, forming approximately 7 to 15 wt.% of it [7,8]. Currently, the formation of tricalcium aluminate during sintering is often affected by various foreign ions. These ions change the resulting structure of tricalcium aluminate and thus also the course of hydration of the cement phase. The most common C_3_A modifiers are sodium and potassium ions in the form of alkalis [9]. They tend to be contained in larger or smaller quantities in raw materials to produce clinker. Their content is measured and controlled, which enables additional correction. In contrast, monitoring the quantitative representation of ions introduced into the material due to the burning of solid alternative fuels is relatively difficult to implement. Nowadays, cement plants use a whole range of secondary fuels for firing clinker in the production of cement. The most commonly used secondary raw materials in incineration are waste oil, used tires, meat, and bone meal or sorted waste, which includes mixed plastics, textiles, textile fibers, carpets, rubber, paper, plastic–paper composite packaging, and chipboard [7,8,9,10].

The most important property of C_3_A affects the processability of cement paste [11,12].

C_3_A occurs in clinker in three modifications depending on the content of alkalis, especially sodium ions. Other oxides that cause C_3_A modification are K_2_O, SiO_2_, MgO, and Fe_2_O_3_ [7,8,13,14]. Pure tricalcium aluminate occurs in a cubic structure. Modification with Na_2_O in particular changes the cubic structure to an orthorhombic structure, monoclinic structure, and two metastable structures, i.e., tetragonal (high-temperature polymorph) and a weakly crystalline form, the so-called “proto-C_3_A”. The reactivity of the given structures is different and so is their course of hydration [15,16,17].

One calcium ion is replaced by two sodium ions. One sodium ion takes the place of the original Ca^2+^ and the other is in the center of the (Al_6_O_18_)^18−^ ring. The general formula of C_3_A modified by sodium ions can be written as Na_2x_Ca_3−x_Al_2_O_6_ where x denotes the amount of calcium ions substituted by sodium ions. As x increases from 0 to 0.25, the crystal system changes from cubic to orthorhombic and then to monoclinic at room temperature. In the C_3_A crystal structure, there are eight sites on the triaxial axes where additional atoms can be inserted. The size of Ca^2+^ and Na^+^ ions is similar, and it is therefore possible to state that Na^+^ ions replace Ca^2+^ atoms in their places in the structure [18,19]. Sodium (Na), which replaces calcium (Ca) in the crystal lattice, occupies a space that is about the same size but slightly larger. When calcium is substituted for sodium, the gaps between the atoms are not filled, leading to a gradual change in the crystal structure. As a result, the initially symmetric cubic lattice becomes a less symmetric orthorhombic or monoclinic [20,21].

Tricalcium aluminate is formed by a solid–solid reaction between CaO and Al_2_O_3_ indirectly through the phases, with various ratios between CaO and Al_2_O_3_. Despite long-term application, there is still no consensus regarding the kinetic and mechanistic aspects of its formation [22,23,24].

The preparation of high-purity C_3_A single phases differs considerably from industrial production. In addition to the classic method based on a high-temperature reaction in the solid phase, there are also alternative methods such as the sol–gel method, combustion syntheses, or synthesis using mechanochemical activation.

The literature [25] states that mechanochemical activation can significantly affect the rate of reactions, whereby the most reproducible and consistent results were obtained under conditions of good interparticle contact with controlled pretreatment to define the physical structure. Mechanochemical activation supports the course of reactions in the solid phase, as it increases the specific surface area and local thermal effects. This is a very energy-intensive preparation, in which the individual processes occur at lower temperatures, however, which, on the other hand, makes it possible to reduce the overall temperature and firing time. The course of the reaction is similar to the high-temperature method [26,27].

Finding a suitable procedure for the sol–gel synthesis of calcium–aluminate phases turned out to be somewhat problematic. For the preparation of calcium–aluminate phases, the Pechini method and its modified versions, which also belong to the group of sol–gel methods, have proven themselves. However, these methods are very far from the real conditions for the formation of calcium aluminate phases in Portland clinker [28].

Therefore, this article is devoted to the traditional method of reaction in the solid phase, which represents the oldest method used for the preparation of the calcium–aluminate phase, but it is still the most frequently used nowadays. In this method, thorough homogenization of the raw materials is very important. If the starting mixture is not homogeneous, the product tends to be contaminated with unreacted starting substances. Preparing a pure phase of the desired composition is often problematic with this method. Unwanted phases are often present in the product. They are present in the product if the reaction does not take place completely or if there are inhomogeneities in the reaction mixture. In the area of inhomogeneities, local phase equilibrium is established, and the corresponding phase is formed.

Previous studies [9,15,16,19,20,21,29,30,31] on calcium–aluminate phases often use a different methodology than the one used in this study. This article is devoted to the preparation of various C_3_A polymorphs when the raw material mixture is doped with sodium, which is added to the raw material mixture before sintering. The aim is to study the formation of individual polymorphs that are formed in parallel. In this way, the preparation procedure presented in this study is close to the real conditions of Portland clinker production. The goal is to prepare pure phases through solid-state synthesis in the highest possible purity.

This article deals with the preparation of various polymorphs of tricalcium aluminate by means of the solid-state method. The aim is to use a simple preparation method that includes mechanical activation of the raw material mixture doped with different Na content and one-step firing to prepare tricalcium aluminate at the highest possible purity.

To evaluate the purity of the prepared C_3_A samples, the Rietveld method was chosen, which involved the Sherrer equation to monitor the crystallinity of the prepared tricalcium aluminates.

## 2. Materials and Methods

### 2.1. Raw Material Mixture Composition

Calcium carbonate (CaCO_3_, p.a. purity, Penta, Praha, Czech Republic), aluminum oxide (Al_2_O_3_, p.a. purity, Penta, Praha, Czech Republic), and sodium carbonate (Na_2_CO_3_, p.a. purity, Penta, Praha, Czech Republic) were used as precursors for the synthesis of tricalcium aluminate. The composition of the raw material mixture for the preparation of tricalcium aluminate was based on already published data [9,30]. The CaCO_3_ and Al_2_O_3_ were mixed in a stoichiometric ratio of 3 mol CaO and 1 mol Al_2_O_3_, which corresponds to the cubic C_3_A modification. Stabilization with sodium (added in the form of sodium carbonate) is necessary to obtain the orthorhombic and monoclinic forms. The content of Na_2_O added to the raw material mixture for the formation of orthorhombic and monoclinic C_3_A is shown in Table 1.

In order to ensure a sufficient amount of Na, which suffers from the relatively high volatility of sodium at high temperatures and resulting losses during sintering, Na_2_CO_3_ was added to the calculated stoichiometric ratio of CaO and Al_2_O_3_.

### 2.2. Tricalcium Aluminate Preparation

Milling and homogenization of the raw material mixture were carried out through high-energy wet milling in a planetary mono mill (Pulverisette 6, Fritsch, Idar-Oberstein, Germany). A 40 mL steel capsule and 25 steel balls with a diameter of 20 mm were used. The basic ratio of dry ingredients and water was 30 g of the raw material mixture and 30 g of water. The water/powder ratio of 1.0 was used. The milling mode of 350 rpm for 15 min was selected. A Malvern Mastersizer 2000 laser granulometer (Malvern Panalytical Ltd., Malvern, UK) with a hydro 2000 G fluid dispersion unit, using 2- 2-isopropanol as the dispersing agent was used for determining the granulometry. After wet milling, all samples were dried in a laboratory oven (Binder C170, Binder GmbH., Tuttlingen, Germany) at a temperature of 105 °C for 24 h. The slurry spontaneously formed solid nodules during the drying process, which were subsequently placed in 30 mL platinum crucibles. The sintering process was carried out in a high-temperature furnace (2017 S, Clasic CZ s.r.o., Řevnice, Czech Republic). The firing mode was designed so that the carbonates first decompose at a temperature of 1000 °C for 1 h and then, the synthesis itself takes place in the solid phase at a temperature of 1300 °C for 2 h. The heating rate was 8 °C/min. At the end of the firing mode, the crucibles were removed from the furnace and immediately cooled by a stream of cold air to room temperature. The sintered products were subjected to milling in a vibratory disc mill (RS 200, Retsch, Haan, Germany) at 1000 rpm for 20 s. After that, 5 g of sintered products in the form of powder was milled to the required fineness for X-ray analysis in a mill (McCrone Micronising Mill, Glen Creston, London, UK) for 150 s in 15 mL of isopropanol.

### 2.3. X-ray Diffraction Analysis

The XRD analysis was performed using a multifunctional diffractometer (XRD, Empyrean, PANalytical B.V., Almelo, The Netherlands). The Θ-Θ reflection Bragg–Brentano para focusing geometry device is equipped with a Cu anode (λ = 1.54184 Å), programmable divergence slits, and a PIXcel3D detector(Empyrean, PANalytical B.V., Almelo, The Netherlands) with 255 active channels, with an accelerating voltage of 45 kV, a beam current of 40 mA, and a diffraction angle of 2 Θ in the range from 5 to 60° with a step size of 0.013° and 38 s per step. The total time per scan was 12 min. Each sample was measured four times, and the scans were next simply summed. The polymorphic purity of the samples was verified via XRD using the Rietveld method. The ICSD database (released in 2012) was used to qualitatively analyze the diffraction patterns. HighScore plus software (3.0e, PANalytical B.V., Almelo, The Netherlands) was used to identify the individual phases, perform their quantification, and determine the amount of the amorphous phase.

### 2.4. Tricalcium Aluminate Crystallinity

The size of the crystallites was evaluated on a selected diffraction line with the major crystallographic direction. Calculation of the crystallite size is based on the measurement of full width at half maximum (FWHM) via the Scherrer equation [32] with Warren correction [33]; see Equation (1):

(1) $$L=\frac{K\cdot \lambda }{\cos\theta }\cdot \frac{1}{\beta }=\frac{K\cdot \lambda }{\cos\beta }\cdot \frac{1}{\sqrt{B^{2}-b^{2}}}$$

*L* is the crystallite size, *K* is the Scherrer constant (the value of 0.89 was used), *λ* = *K*_α1_ is the wavelength of X-ray radiation, *θ* is the diffraction angle, *B* is the FWHM, and *b* is the FWHM of the size/strain standard used (lanthanum hexaboride).

**Figure 1:**
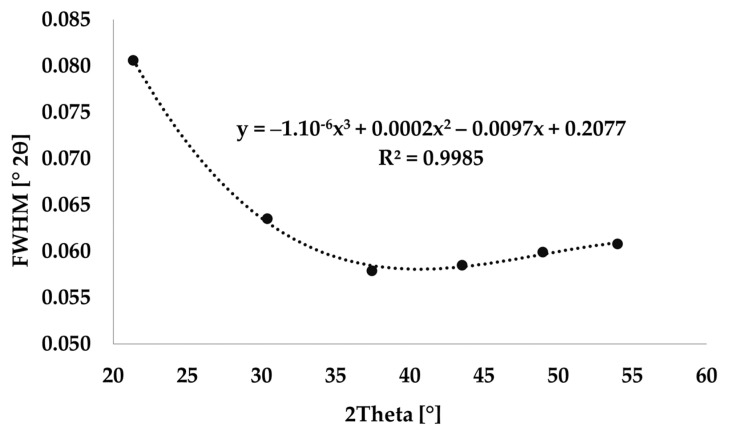
Instrumental broadening measured using LaB_6._

The instrument broadening was defined using the LaB_6_ standard. LaB_6_ peaks were determined in the positions identical to the selected diffraction lines using HighScore plus software, see Figure 1. The FWHM values of LaB_6_ were calculated from Equation (1) [34].

## 3. Results and Discussion

### 3.1. Tricalcium Aluminate Preparation

**Figure 2:**
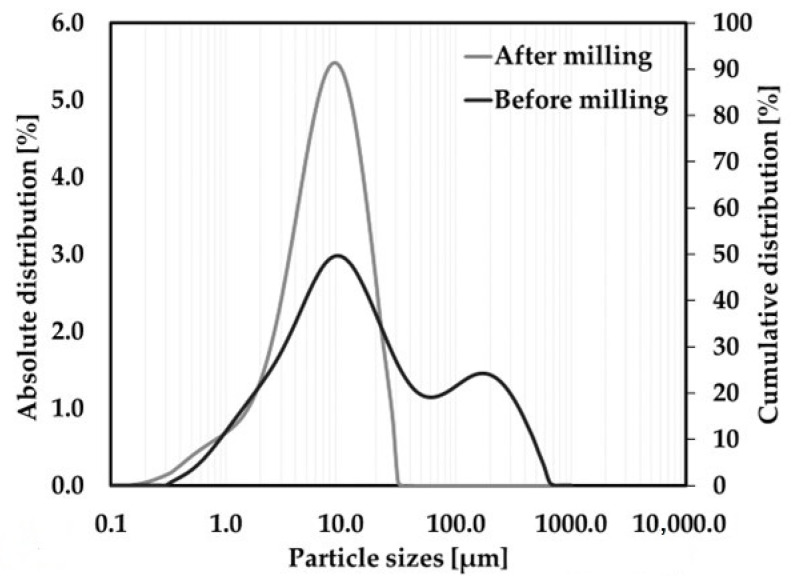
Particle distribution (PSD) of the raw material mixture.

The distribution of particles after the homogenization of raw materials through high-energy milling was determined using laser granulometry; see Figure 2.

The median particle size of the raw material mixture before homogenization in the Pulverisette 6 laboratory mill (Pulverisette 6, Fritsch, Idar-Oberstein, Germany) was 12.3 μm. After 15 min of milling at 350 rpm, the raw material mixture was refined with a median particle size of 6.8 μm.

### 3.2. X-ray Diffraction Analysis

Table 2 shows the quantitative mineral composition of each sample. The results of the Rietveld analysis are presented in Appendix A.

The majority of C_3_A phases were identified on the diffraction patterns: C_3_A cubic (ICSD 00-038-1429), C_3_A orthorhombic (ICSD 1880), and C_3_A monoclinic (ICSD 100221). The mineral mayenite (ICSD 00-009-0413), lime (ICSD 00-043-1001), and portlandite (ICSD 00-004-0733) as minor phases were also identified.

**Figure 3:**
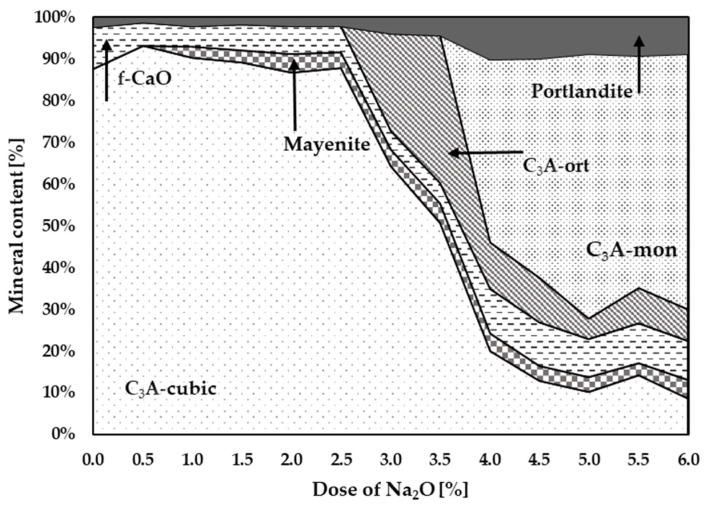
Graphical representation of individual mineral contents at different Na contents.

Figure 3 graphically shows the effect of Na doses on the change in the C_3_A polymorphism. The presence of higher Na content is a basic prerequisite for the formation of non-cubic C_3_A phases. It can be seen in Table 2 and Figure 3 that at least 2.5–3% Na is needed for the formation of non-cubic phases of tricalcium aluminate. At Na content of 3 to 4%, the formation of the orthorhombic C_3_A is supported. At higher doses of Na above 3.5–4%, the monoclinic phase C_3_A is formed.

The presence of portlandite is caused by the very rapid hydration of lime due to air moisture. The reason for the higher content of lime, i.e., portlandite, can be determined according to another study [31], with the release of free lime during the formation of a solid solution of C_3_A with sodium ions calculated according to the following equation:

(2) $$3{Ca}_{3}{Al}_{2}O_{6}+{Na}_{2}O↔{Na}_{2}{Ca}_{8}{Al}_{6}O_{18}+CaO$$

The synthesized cubic, Na-substituted orthorhombic, and monoclinic C_3_As were evaluated as mixtures, indicating that accurate quantification is very difficult to perform.

**Figure 4:**
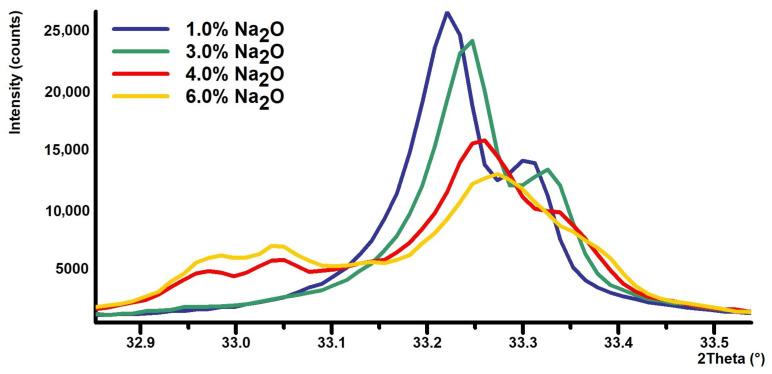
Portion of XRD powder patterns: 1.0% Na_2_O (cubic C_3_A), 3.0% Na_2_O (cubic + orthorhombic C_3_A), 4% Na_2_O (cubic + orthorhombic + monoclinic C_3_A), and 4% Na_2_O (mainly monoclinic C_3_A).

Identification and quantification of cubic C_3_A is generally easy because the diffraction lines are well defined and clearly match the reference pattern. Substitution of Na, however, causes significant difficulties because the pattern of cubic and orthorhombic or monoclinic forms significantly overlaps, see Figure 4. Therefore, it is quite difficult to clearly and accurately fit the individual diffraction lines of cubic, orthorhombic, and monoclinic C_3_A using the XRD technique.

#### Unit Cell Volume

**Figure 5:**
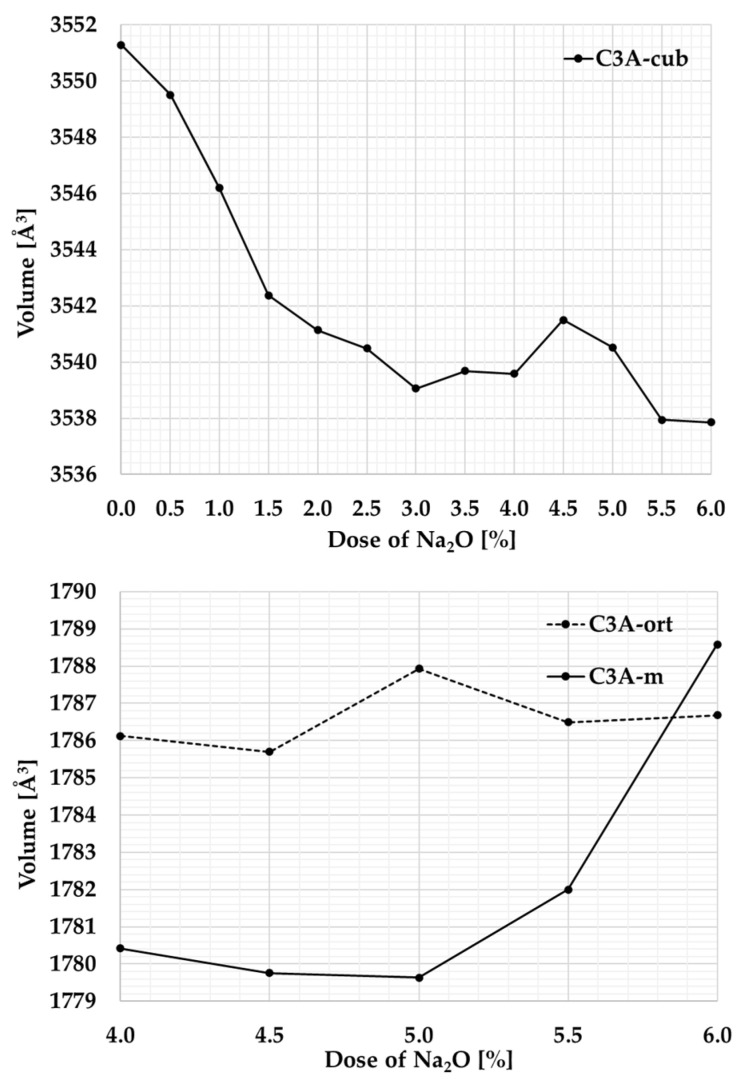
Graphical representation of changes in the unit cell volume at different Na contents.

The Rietveld method was also used in studying the crystal structure to provide more detailed information about the crystal lattice of the individual polymorph phases of tricalcium aluminate. Table 3 shows the unit cell volumes determined using the Rietveld method, graphically in Figure 5.

Sodium (Na), which replaces calcium (Ca) in the crystal lattice, occupies a space that is slightly larger. When sodium is replaced by calcium, the spaces between the atoms are not filled, leading to a gradual change in the crystal structure. As a result, the initially symmetric cubic lattice becomes a less symmetric orthorhombic and subsequently monoclinic structure. At Na content of 3%, the mixture contains cubic C_3_A with a unit cell volume of around 3545 Å^3^ and orthorhombic C_3_A with almost half the volume of the unit cell.

### 3.3. Tricalcium Aluminate Crystallinity

To supplement more detailed information and connections with the origin and development of the C_3_A polymorphism, the development of crystallinity was monitored.

**Figure 6:**
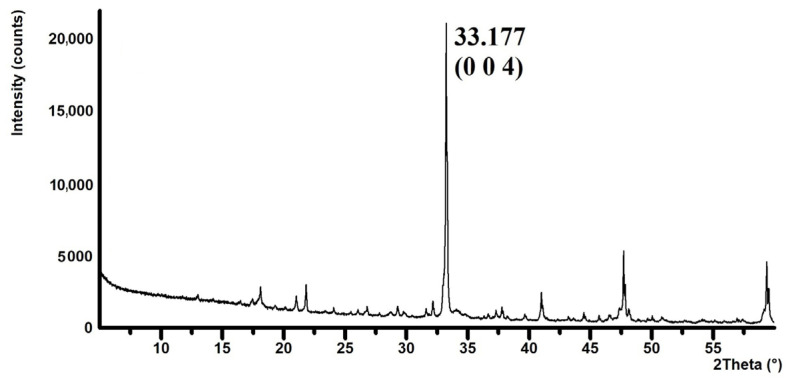
Details of the reflection of the selected crystallographic plane (0 0 4) on the XRD pattern.

The size of the crystallites was evaluated in the normal to appropriate crystallographic plane that had the main crystallographic direction and was sufficiently intense. A distinct crystallographic plane determined by the Miller indices “hkl”: 004 was chosen, which appears on the diffraction pattern at the position 2θ: 33.177°; see Figure 6.

**Figure 7:**
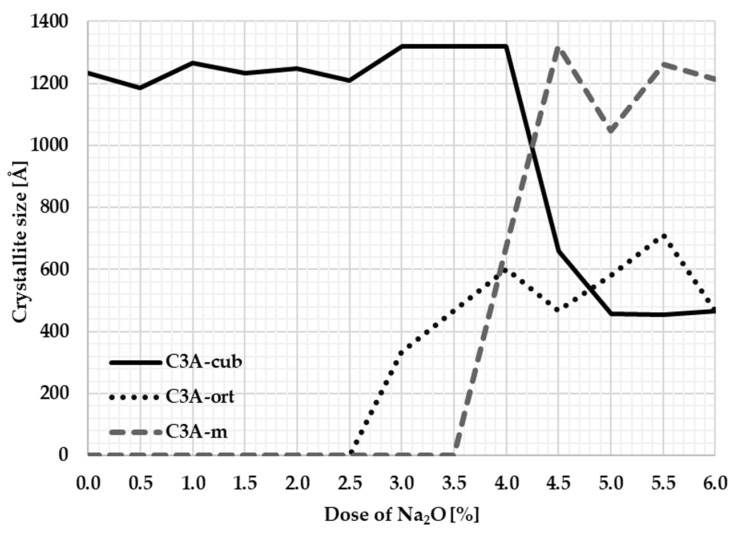
Dependence of crystallite size on Na content.

The Sherrer method was used to calculate the crystallite size. Table 4 and Figure 7 graphically show the crystallite sizes for individual samples.

The course of the crystallite size corresponds to the changes in the unit cell during the transformations of the individual crystal structures. Based on the calculation of the size of the crystallites, it can be stated that the most significant changes in crystallinity occur at Na_2_O content of between 3 and 4 percent when there is a fundamental polymorphic change from cubic to orthorhombic. The average crystallite size of cubic C_3_A is around 1200 Å. When the sodium ions are incorporated into the C_3_A cubic structure, the size of its crystallites rapidly decreases to an average value of 466 Å. At 4% sodium content, the orthorhombic structure changes to a monoclinic structure. The average crystallite size of the monoclinic structure is around 1104 Å and approaches the crystallite size of the original cubic C_3_A.

## 4. Conclusions

The results obtained in this study allow the following conclusions to be reached:

- The various polymorphs of C_3_A were prepared through the method of solid-state synthesis and a procedure close to the real conditions of the formation of Portland clinker.
- The presence of multiple polymorphs might be due to the particle size of the raw meal.
- Although powder diffraction has a fundamental limitation in the reliable identification and quantification of C_3_A polymorphs, it is the only accessible approach.
- The stability of cubic tricalcium aluminate is up to 2.5% Na content.
- The formation of orthorhombic tricalcium aluminate using solid-state synthesis, as described, requires Na content of at least 3–3.5% Na.
- The transition from an orthorhombic structure to a monoclinic structure takes place at 4–4.5% Na content.
- The most significant changes in crystallinity occur at 3–4% Na content.
- There are no clearly defined boundaries for the existence of individual C_3_A phases, these phases arise at the same time and overlap each other in the areas of their formation at different Na doses.

## Figures and Tables

**Table 1:** Composition of Na-substituted C_3_A raw material mixture.

Label	CaCO_3_ [g]	Al_2_O_3_ [g]	Na_2_CO_3_ [g]	Total
0.0% Na_2_O	22.40	7.60	0.00	30.000
0.5% Na_2_O	22.40	7.60	0.26	30.260
1.0% Na_2_O	22.40	7.60	0.51	30.510
1.5% Na_2_O	22.40	7.60	0.77	30.770
2.0% Na_2_O	22.40	7.60	1.03	31.030
2.5% Na_2_O	22.40	7.60	1.28	31.280
3.0% Na_2_O	22.40	7.60	1.54	31.540
3.5% Na_2_O	22.40	7.60	1.8	31.800
4.0% Na_2_O	22.40	7.60	2.05	32.050
4.5% Na_2_O	22.40	7.60	2.31	32.310
5.0% Na_2_O	22.40	7.60	2.57	32.570
5.5% Na_2_O	22.40	7.60	2.82	32.820
6.0% Na_2_O	22.395	7.605	3.08	33.080

**Table 2:** Rietveld refinement quantified phase composition of the samples.

Na_2_O [%]	C_3_A-c	C_3_A-o	C_3_A-m	Mayenite	f-CaO	Portlandite	Total	GOF
0.0	89.7	0.0	0.0	0.0	8.0	2.3	100.0	2.55
0.5	94.4	0.0	0.0	0.0	4.5	1.2	100.1	2.48
1.0	92.1	0.0	0.0	2.7	3.2	2.0	100.0	2.54
1.5	90.5	0.0	0.0	3.1	4.7	1.6	99.9	2.49
2.0	88.8	0.0	0.0	4.4	4.6	2.2	100.0	2.59
2.5	89.9	0.0	0.0	4.0	4.0	2.2	100.1	2.60
3.0	66.9	24.3	0.0	4.0	0.8	4.0	100.0	2.30
3.5	52.9	36.8	0.0	5.0	0.6	4.6	99.9	2.48
4.0	22.2	12.2	48.9	4.8	0.6	11.3	100.0	1.69
4.5	14.3	12.0	58.3	3.8	0.7	10.9	100.0	1.68
5.0	11.0	5.6	69.5	4.0	0.4	9.5	100.0	2.27
5.5	15.7	9.2	61.3	3.0	0.5	10.2	99.9	2.19
6.0	9.5	8.4	67.0	4.7	0.7	9.6	99.9	2.47

**Table 3:** Unit cell volume of cubic, orthorhombic, and monoclinic C_3_A.

Na_2_O [%]		Volume [Å^3^]		Volume [Å^3^]		Volume [Å^3^]
0.0	C_3_A-cub	3551.29	C_3_A-ort	0.00	C_3_A-m	0.00
0.5		3549.51		0.00		0.00
1.0		3546.20		0.00		0.00
1.5		3542.38		0.00		0.00
2.0		3541.13		0.00		0.00
2.5		3540.49		0.00		0.00
3.0		3539.06		1778.56		0.00
3.5		3539.69		1779.86		0.00
4.0		3539.58		1786.13		1780.42
4.5		3541.50		1785.70		1779.76
5.0		3540.52		1787.93		1779.63
5.5		3537.95		1786.49		1782.00
6.0		3537.86		1786.68		1788.58

**Table 4:** Crystallite size of cubic, orthorhombic, and monoclinic C_3_A (L Vol-FWHM).

Na_2_O [%]		L [Å]		L [Å]		L [Å]
0.0	C_3_A-cub	1233.6	C_3_A-ort	0.0	C_3_A-m	0.0
0.5		1184.3		0.0		0.0
1.0		1264.8		0.0		0.0
1.5		1233.6		0.0		0.0
2.0		1249.0		0.0		0.0
2.5		1208.4		0.0		0.0
3.0		1320.5		332.7		0.0
3.5		1320.5		466.2		0.0
4.0		1320.5		602.2		680.0
4.5		659.3		467.9		1320.5
5.0		457.2		580.5		1047.8
5.5		455.6		711.2		1262.5
6.0		467.4		467.4		1212.5

**Figure A1:**
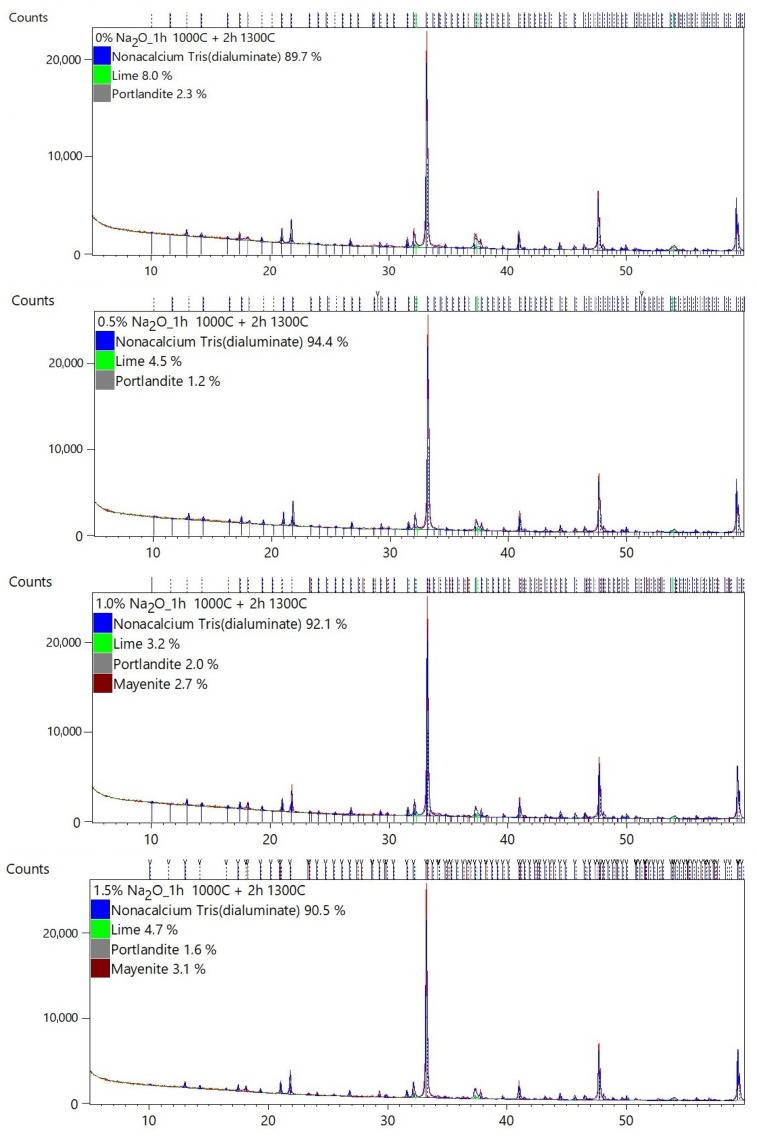

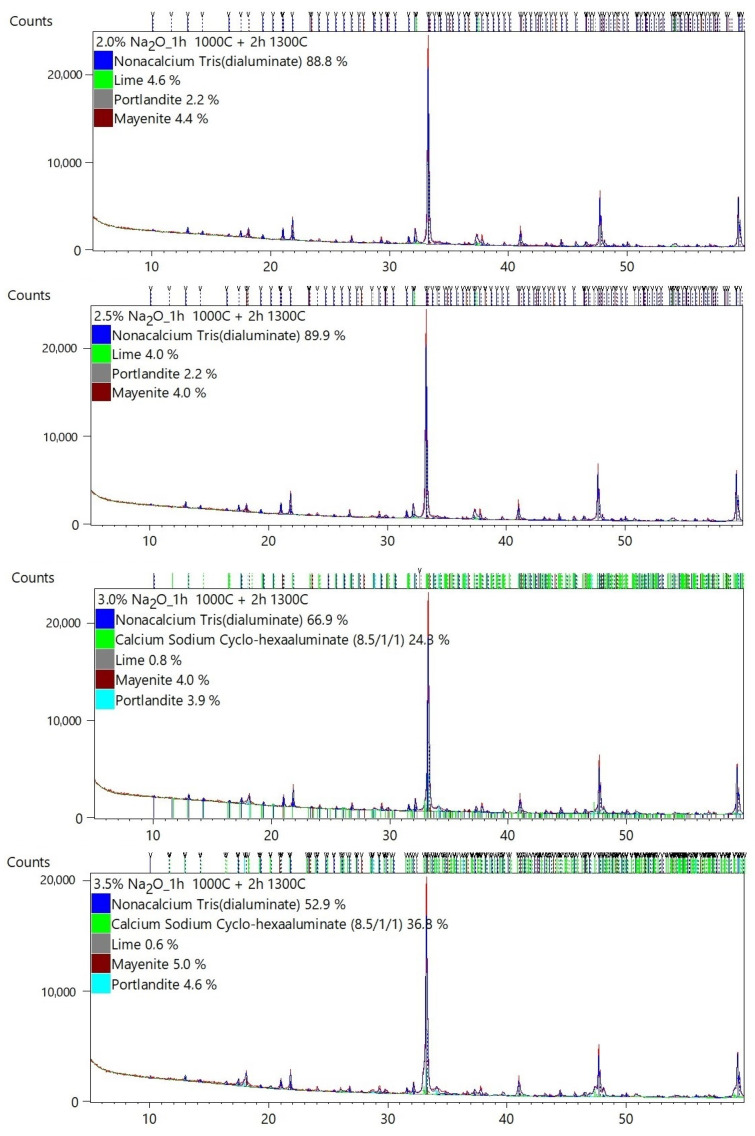

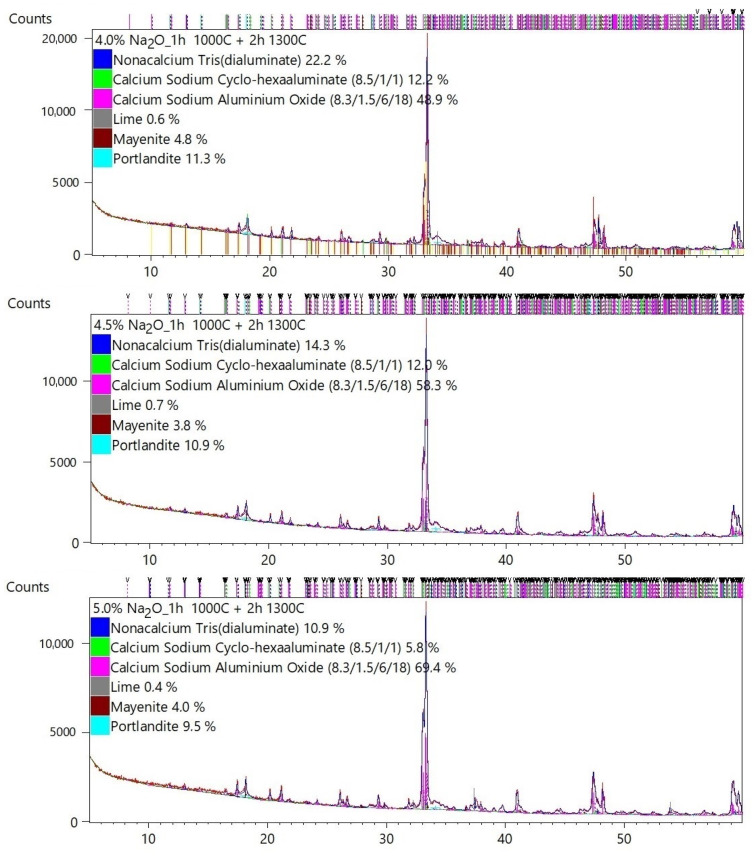

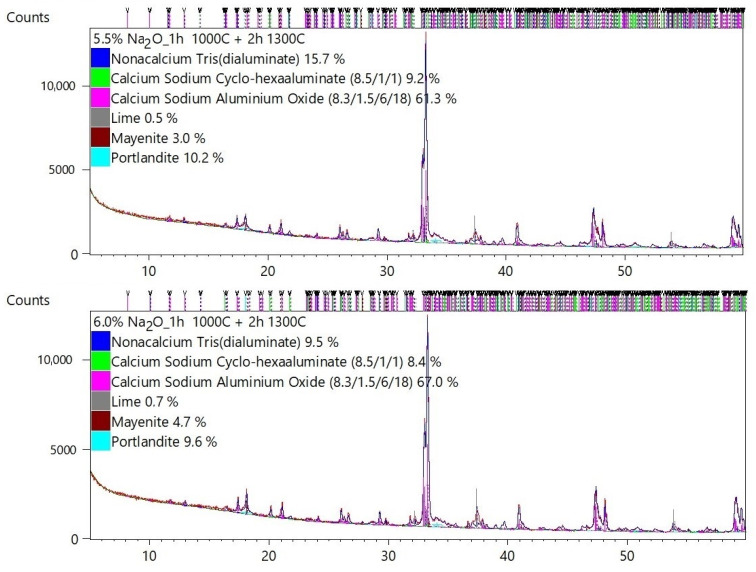
Rietveld analysis results.

## Data Availability

Data are contained within the article.
